# Spinocerebellar Ataxia Type 2: Clinicogenetic Aspects, Mechanistic Insights, and Management Approaches

**DOI:** 10.3389/fneur.2017.00472

**Published:** 2017-09-11

**Authors:** Luis C. Velázquez-Pérez, Roberto Rodríguez-Labrada, Juan Fernandez-Ruiz

**Affiliations:** ^1^Centre for the Research and Rehabilitation of Hereditary Ataxias, Holguín, Cuba; ^2^Medical University of Holguín “Mariana Grajales”, Holguín, Cuba; ^3^Physical Culture School, University of Holguin “Oscar Lucero”, Holguín, Cuba; ^4^Department of Physiology, Medicine School, UNAM, Cuernavaca, Mexico; ^5^Psychology School, Universidad Veracruzana, Xalapa, Mexico

**Keywords:** spinocerebellar ataxia type 2, ataxin-2, polyglutamine expansions, hereditary ataxias, autosomal dominant cerebellar ataxias

## Abstract

Spinocerebellar ataxia type 2 (SCA2) is an autosomal dominant cerebellar ataxia that occurs as a consequence of abnormal CAG expansions in the *ATXN2* gene. Progressive clinical features result from the neurodegeneration of cerebellum and extra-cerebellar structures including the pons, the basal ganglia, and the cerebral cortex. Clinical, electrophysiological, and imaging approaches have been used to characterize the natural history of the disease, allowing its classification into four distinct stages, with special emphasis on the prodromal stage, which is characterized by a plethora of motor and non-motor features. Neuropathological investigations of brain tissue from SCA2 patients reveal a widespread involvement of multiple brain systems, mainly cerebellar and brainstem systems. Recent findings linking ataxin-2 intermediate expansions to other neurodegenerative diseases such as amyotrophic lateral sclerosis have provided insights into the ataxin-2-related toxicity mechanism in neurodegenerative diseases and have raised new ethical challenges to molecular predictive diagnosis of SCA2. No effective neuroprotective therapies are currently available for SCA2 patients, but some therapeutic options such as neurorehabilitation and some emerging neuroprotective drugs have shown palliative benefits.

## Introduction

Spinocerebellar ataxias (SCAs) comprise a large heterogeneous group of autosomal dominant cerebellar ataxias caused by a large variety of genetic defects including repeat expansions, conventional mutations, and large rearrangements in genes (1, 2). SCAs have been classified into at least 43 subtypes depending on their genetic locus. Among them, SCA type 2 (SCA2) is the second most common disorder and one of the most severe subtypes (3, 4). The disease is caused by the abnormal expansion of *Cytosine–Adenine–Guanine* (CAG) repeats in a coding region of the *ATXN2* gene (12q23-q24.1), which leads to the expression of abnormally long polyglutamine (polyQ) sequences in the homonymous protein (5). polyQ-expanded ataxin-2 exhibits toxic properties and loses its biological functions causing dysfunction and death of a large population of neurons in the cerebellum, brainstem, spinal cord, and brain cortex, which lead the mechanisms of the progressive cerebellar syndrome, including the extra-cerebellar features, which clinically characterizes the disease (3, 6). Currently, converging evidence from clinical, electrophysiological, and imaging approaches indicate that the toxic damage starts years before the ataxic onset, with a SCA2 prodromal stage characterized by subtle motor and unspecific non-motor features, preceding the clinical diagnosis by up to 15 years (7–11).

Although incurable, SCA2 is not an untreatable disease. Some therapeutic options such as physiotherapy and neuroprotective drugs have beneficial effects in patients. Nevertheless, meaningful clinical trials recruiting large numbers of patient with similar genetic and environmental backgrounds, preferably subjects in prodromal or early disease stages are required to confirm the efficacy of these and future promising therapies (3, 6).

The present review addresses the main epidemiological, phenotypic, and genotypic features of SCA2, as well as the diagnostic and therapeutic approaches.

## Epidemiology

Global epidemiological data show that SCA2 has a wide geographical distribution across the world, being the second most common subtype of autosomal dominant cerebellar ataxia at worldwide, after only to SCA3 (Machado–Joseph disease); nevertheless in some parts of the world, it represents the most common subtype and, in other regions, it is a less frequent disorder as compared to other SCAs (1).

**Figure 1:**
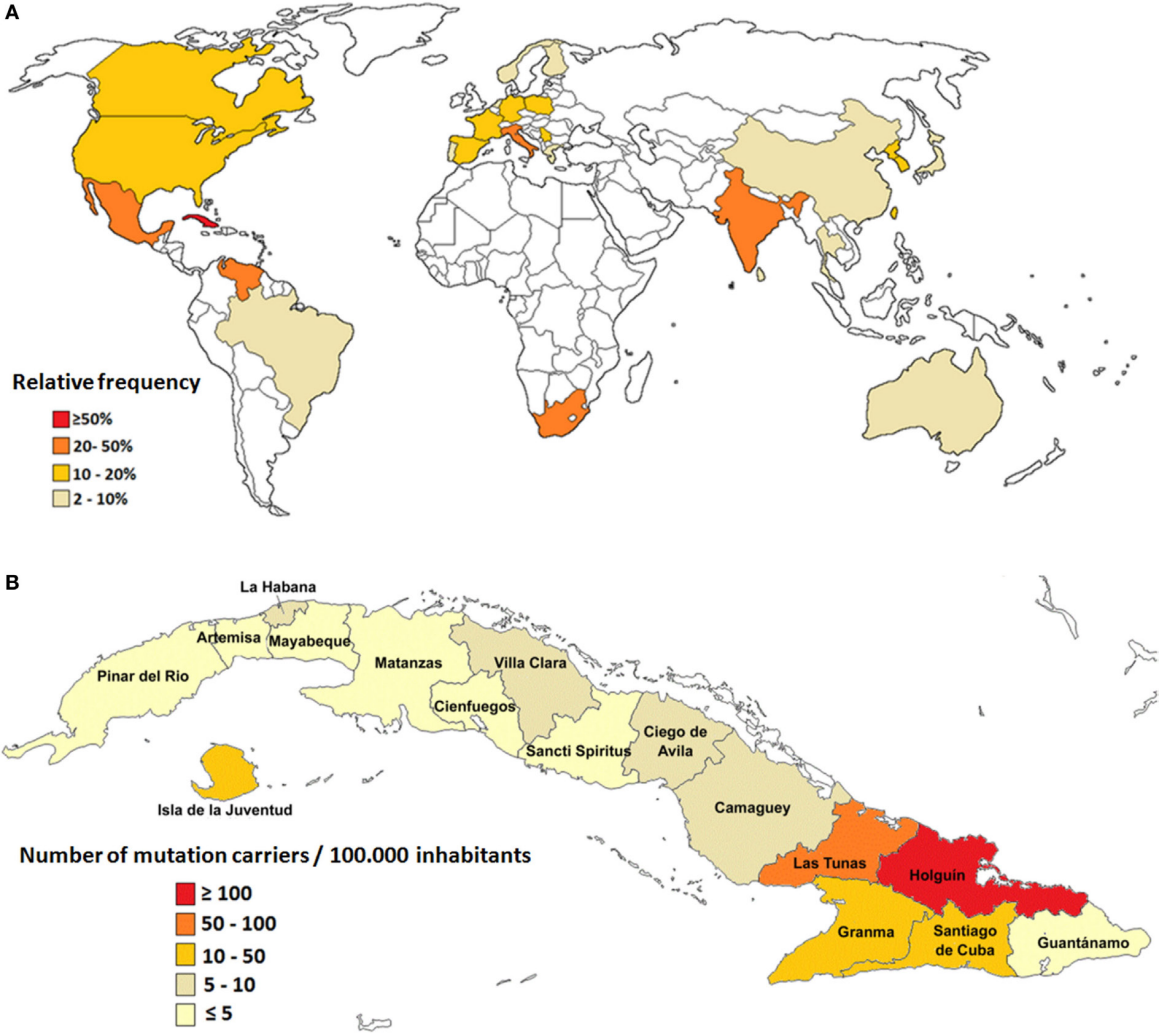
Epidemiological features of spinocerebellar ataxia type 2 (SCA2). **(A)** Relative frequency of SCA2 around the world. **(B)** Prevalence rates of SCA2 in Cuba.

However, the small number of population surveys, most of them performed in isolated geographical regions, hinder the unbiased evaluation of global prevalence for SCA2 (1). Large populations of SCA2 families have been described in Mexico (12), South Africa (13), India (14), Italy (15), and Venezuela (16); however, the largest prevalence rates are found in Cuba due to a prominent founder effect (17, 18) (Figure 1A).

In Cuba, 160 SCA2 families have been reported, which involve almost 800 patients, 3,500 first-degree “at-risk” descendants, and 6,500 second-, third-, or fourth-degree “at-risk” individuals. The nationwide prevalence rate is 6.57 cases per 100,000 inhabitants for patients and 28.51 mutation carriers per 100,000 inhabitants (patients and preclinical carriers). In addition, Cuba has the highest frequency of normal large alleles of the *ATNX2* gene, which represents unstable pre-mutated variants considered as reservoirs for mutated alleles leading to the continuous rise of new SCA2 cases (19, 20).

Inside the island, the highest case frequency is observed in the northeastern region, with remarkable prevalence in the Holguin province, with 40.18 cases and 182.75 carriers per 100,000 inhabitants (Figure 1B). Within Holguin province there are areas with extremely high prevalence rates, such as Baguanos and Cacocum municipalities, where the annual incidence rate is near 18 cases per 100,000 inhabitants (17).

## Phenotypical Features

Neurological and electrophysiological assessments of SCA2 patients have enabled a comprehensive characterization of disease phenotype since its early stages, suggesting that SCA2 is a multisystem disorder. Therefore, the clinical picture of the disease consists on a wide range of motor and non-motor features underlying the involvement of cerebellum, brainstem, brain cortex, basal ganglia, spinal cord, and peripheral nerves among other structures.

### Motor Cerebellar Manifestations

The clinical hallmark of all SCAs is the cerebellar syndrome (1, 2, 8). SCA2 is characterized by a broad group of progressive features, including gait ataxia, postural instability, cerebellar dysarthria, dysmetria, and dysdiachokinesia, whereas hypotonia is not so common. Usually the presenting symptom of the cerebellar syndrome in SCA2 is the gait ataxia (97%), while a few patients referred to cerebellar dysarthria as their first symptom. The age of onset of the cerebellar syndrome in SCA2 is variable, although in most subjects, it appears in the second or third decade of life. Similar to other SCAs caused by polyQ expansions, the age of onset is closely correlated to the expanded CAG repeats in the *ATXN2* gene, which explain between 60 and 80% of its variability (3, 6).

Recently, in an 8-year longitudinal study, Jacobi et al. (21) compared the progressive patterns of cerebellar syndrome in various European cohorts of SCAs. Similar to SCA1, SCA3, and SCA6, the progression of ataxia in SCA2, as measured by the scale for the assessment and rating of ataxia (SARA), was best fitted with a linear model. The mean annual progression of SARA score in SCA2 patients was 1.49 ± 0.07, which was only significantly surpassed by the SCA1 progression rate (2.11 ± 0.12) and comparable with SCA3 (1.56 ± 0.08). In SCA6, the progression was notably slower than the other subtypes (0.80 ± 0.09). Similar progression patterns of cerebellar syndrome had been obtained previously in an U.S. cohort (22).

Factors modulating the cerebellar syndrome progression in SCA2 are not consistent among distinct studies. The aforementioned longitudinal study identified the lower age at onset, whereas Tezenas du Montcel and coworkers identified other modifying factors such as the age at inclusion, gender and number of CAG repeats in the unexpanded alleles (23). In addition, a recent follow-up study in 30 Cuban patients disclosed a role of the expanded CAG repeats as significant influencing factor in the progression of SCA2 cerebellar abnormalities (24).

### Non-Cerebellar Manifestations

#### Oculomotor Disturbances

The most salient feature of oculomotor disturbance in SCA2 is the slowing of horizontal saccadic eye movements. The bedside clinical examination of saccades reveals this oculomotor sign in more than 80% of cases, being above 90% in the Cuban SCA2 population (25). However, electronystagmographical assessments can detect the saccade slowing in almost all SCA2 patients (98%), which defines this oculomotor manifestation as pathognomonic sign of SCA2 (24, 26, 27).

Maximal saccade velocity is inversely correlated to expanded CAG repeats in the expanded *ATXN2* alleles, suggesting the strong genetic control of this disturbance and identifying it as the main endophenotype of the SCA2, with high usefulness as sensitive biomarker for the study of polyQ toxicity (26). In addition, SCA2 patients exhibit significant increases in saccade latency (28) and saccade dysmetria, which together with saccade slowing deteriorates over time (24). Other oculomotor features include abnormal smooth pursuit, reduced vestibulo-ocular reflex, and ophtalmoplegia, whereas nystagmus is very rare due to impaired ability to produce saccadic corrective phases (27, 29).

#### Signs of Corticospinal Tract (CST) Dysfunctions

The main clinical signs of CST dysfunctions in SCA2 include extensor plantar response (31%), hyperreflexia (13.2%), and spasticity (8.9%) (30). This clinical picture is supported by increases in motor threshold, cortical silent period, and central motor conduction time of motor evoked potentials obtained through non-invasive transcranial magnetic stimulation (31, 32), as well as by the observation of degeneration of motor cortex neurons (4).

#### Signs of Lower Motor Neuron Degeneration

The damage to alpha motor neurons or motor neuronopathy in SCA2 patients results in several symptoms and signs assessed by both bedside clinical examination and electromyography (EMG) (3, 6). These features become detectable after the upper motor neuron involvement and include fasciculations (38.3%) and distal amyotrophy (22.5%). EMG assessments revealed some denervation signs such as fibrillations, positive sharp waves, and fasciculations. The motor unit potentials have increased amplitudes, whereas the F wave showed increased latencies and decreased persistence (33). Currently, the recognition of signs of lower motor neuron degeneration in SCA2 acquires additional clinical and pathogenetic relevance given the known relationship between the intermediate alleles of *ATXN2* gene and increased risk of amyotrophic lateral sclerosis (ALS) (34). The link between the ATXN2 gene and lower motor neuron degeneration was also demonstrated by Jardim and coworkers in 2003, who detected that in SCA3 patients the CAG repeat length of the ATXN2 gene was associated with fasciculations (35).

#### Eextrapyramidal Features

Movement disorders beyond cerebellar ataxia are prominently observed in some forms of SCAs, being SCA2 is one of the most affected subtypes. In a European series of 163 SCA2 patients, resting tremor was observed in 14.9% of cases, followed by dystonia (14.2%), myoclonus (13.7%), rigidity (7.4%), and chorea/dyskinesia (6.8%). In this cohort, the authors identified a significant influence of expanded CAG repeats on some of these manifestations (30).

The parkinsonian phenotype of SCA2 is levodopa responsive, and it is usually associated to later age at onset and a shorter CAG repeat expansion with CAA interruptions stabilizing the combined repeat tract. This phenotype seems to occur more preferentially in Asians populations (36). Molecular mechanisms underlying parkinsonism caused by *ATXN2* expansions are still unclear. Nevertheless, the prevention of somatic mosaicism of CAG stretch by the triplet repeats interruptions could result in parkinsonian features instead ataxia (37). In addition; neuropathological findings suggest that SCA2-related parkinsonism results from Lewy related α-synuclein pathology in the brainstem (38).

#### Peripheral Neuronopathy

The involvement of peripheral nerves in SCAs is not rare, and in SCA2 patients, it could appear in up to 90% of cases as a mixed sensori-motor peripheral neuronopathy with higher predominance of axonal involvement signs (39–41). The features of sensory peripheral neuronopathy in SCA2 are supported by the early cell loss of the dorsal ganglia root (42), and they include hypo/areflexia, paresthesia, hypoesthesia, and hypo-pallesthesia. Motor symptoms are late and usually related to muscle atrophy and distal weakness (40), resembling the motor neuron disease.

Nerve conduction studies have demonstrated a marked reduction of the amplitudes of sensory potentials as signs of axonal damage (40), which show a significant and CAG-dependent progression across time (41). This last feature is followed by secondary signs of demyelination (40).

#### Painful Muscle Cramps

Painful disabling muscle cramps are common in SCA2 patients. Cramps are reported by 88% of the patients and usually affect the lower limbs, followed by abdominal and trunk muscles (25). The highest incidence of these symptoms occurs during the sleep causing frequent awakenings in the patients. The age at onset of muscle cramps is inversely correlated to the CAG repeat size, accounting for a 76% of the variability (9). Although the physiopathological mechanisms underlying muscle cramps in SCAs are not fully understood, it has been proposed that they result from motor neurons distal portions hyper-excitability states caused by collateral sprouting processes after subtle axonal damage in these fibers (43).

#### Sleep Disorders

Spinocerebellar ataxia type 2 patients with most important sleep disorders include restless legs syndrome (RLS), periodic legs movements syndrome (PLMS), REM sleep behavior disorders (RBD), insomnia, and nocturnal leg cramps. Polysomnographic findings reveal a notably abnormal sleep architecture with significant reductions of sleep efficiency, N2 stage percentage, and REM sleep percentage, as well as increased arousal index (44, 45).

Restless legs syndrome and PLMS are present in 25 and 38% of SCA2 patients, respectively, and are associated to larger disease duration likely as a result of brain dopaminergic hypoactivity (44). Moreover, REM sleep pathology in SCA2 is closely associated to cerebellar syndrome severity and it is characterized by reduced REM sleep stage percentage and REMs density as well as increased EMG activity [REM sleep without atonia (RWA)]. The study of RWA percentages acquire additional significance because this alteration is considered a subclinical marker of RBD and it is notably influenced by the CAG expansion size, suggesting the particular vulnerability to polyQ toxicity in the REM sleep generator sites. In addition, other sleep findings such as nocturnal muscle cramps, insomnia, and central sleep apnea have been described in SCA2 (44).

#### Cognitive Decline

Cognitive performance of SCA2 patients is characterized by early frontal-executive dysfunctions, verbal memory impairments and attentional deficits. Although some studies have reported a high frequency of demented patients, the disease does not necessarily evolve in full blown dementia in the majority of cases (46, 47). Frontal-executive deficits in SCA2 include impaired working memory, inhibitory control, strategy, planning, and rule acquisition, which could result from the involvement of frontal cortex and subcortical structures, such as basal ganglia, as well as from the disruption of cerebro-cerebellar-cerebral loops (47, 48). These cognitive functions have been assessed through neuropsychological and electrophysiological approaches, in particular using the antisaccadic paradigm, which identifies this cognitive disturbance in almost 70% of cases and offers significant evidences on the role of the expanded CAG repeats over the frontal-dependent executive functions in SCA2 patients. In addition, a higher dys-executive deficit is associated with higher severity of cerebellar syndrome suggesting the parallel progression of both features (49). Furthermore, the application of prism adaptation tests in SCA2 patients has revealed reduced visuomotor learning in these subjects, which may be related to attentional, executive, and procedural disturbances (50).

#### Psychiatric Symptoms

Psychiatric manifestations are also common comorbidities in SCAs. For SCA2 patients, the most frequent symptoms include depression and anxiety states, whereas psychosis is rare (51, 52). Depressive symptomatology is reported up to 22% of cases, but only 7% fit the criteria of major depression (53). These features are associated to the ataxia severity, suggesting the role of the patients’ disability perception on the depressive states and the possible involvement of the limbic cerebellum in the posterior lobe (52, 53).

#### Autonomic Disturbances

Dysfunctions of the autonomic nervous system are important clinical components of SCA2 patients. Deficits include urogenital, cardiovascular, gastrointestinal, and thermoregulatory dysfunction as a result of damage of autonomic ganglia and central pathways (54). An study conducted in 97 Cuban SCA2 patients revealed dysautonomic symptoms in 67% of cases. Nocturia and pollakiuria were the most frequent complaints, followed by dysphagia and constipation. Interestingly, those patients carrying larger CAG expansions and higher ataxia severity exhibit more significant dysautonomic disturbances (55).

On electrophysiological assessments, heart rate variability studies in SCA2 patients reveal a cardiovascular autonomic dysfunction in above 60% of individuals, which consists of abnormal responses to deep breathing and Valsalva maneuver, head-up tilt test, standing, hand gripping test, and others. Spectral analyses of resting heart rate variability disclose reductions of high frequency and low frequency power, suggesting the combined involvement of sympathetic and parasympathetic systems in the cardiovascular autonomic dysfunction (56, 57).

#### Olfactory Dysfunction

After initial evidence of olfactory dysfunction in hereditary ataxias (58), Velázquez-Pérez et al. (59) assessed olfactory function of SCA2 patients, by smell identification an olfactory threshold tests. This study demonstrated a notable pattern of deficits related to impaired olfactory threshold, quality, identification and discrimination. These abnormalities were no associated to expanded CAG repeats, ataxia severity nor disease duration, but the odor identification impairments were directly correlated to global cognitive dysfunctions. Although the pathological substrate of olfactory dysfunctions in SCA2 is not fully understood, these features could result from peripheral and/or central causes. The first ones could be related with the impaired function of the expanded ataxin-2 in the olfactory habituation in the olfactory bulb (60), whereas the central causes might involve the cerebellar damage since some evidences have revealed a role of the cerebellum in olfaction (61). Nevertheless, both hypotheses need to be confirmed by further studies.

### Infantile Phenotype

Although SCA2 is considered as an adult-onset disease, an infantile or pediatric phenotype has been described in some populations as result of large CAG expansions. The clinical picture of SCA2 infantile phenotype includes a severe form of cerebellar involvement accompanied by a set of unusual symptoms and signs such as retinitis pigmentosa, myoclonus-epilepsy, tetraparesis, developmental delay, facial dysmorphism, oculomotor apraxia, vasomotor instability deterioration of expressive language, comprehension and memory deficits, graphomotor skills, and dysarthria, as well as progressive extrapyramidal manifestations, trophic changes, and dysphagia (62–64).

### Prodromal Features

Prodromal SCA2 denotes the stage wherein early functional and clinical features of neurodegeneration are present but are insufficient to define disease, based on a fully evolved cerebellar syndrome (7). The main features of SCA2 prodromal stage include painful muscle cramps (9, 10), oculomotor disturbances such as early saccade slowing and nystagmus (10, 65), symptoms and electrophysiological signs of peripheral neuropathy (9, 10), CST dysfunction (9, 10, 66), sleep disturbances (10, 67) and dysautonomia (10, 68), as well as subtle cognitive decline (10), and olfactory dysfunctions (11). SCA2 prodromal stage is also characterized by subtle motor cerebellar manifestations that have not enough progressed to conclusively diagnose cerebellar syndrome, such as abnormalities of tandem gait (9), postural instability (69), and other subtle coordination deficits assessed by SARA score (70) and prism adaptation task (71).

In SCA2 preclinical carriers, the expanded CAG repeats are notably correlated with the age at onset of muscle cramps, sensory abnormalities, hyperreflexia, and subtle motor cerebellar features (9), as well as with the severity of saccade slowing (65), CST dysfunction (66), and postural instability (69). Moreover, most of SCA2 prodromal features increase significantly in subjects with higher probabilities to be clinically diagnosed by the short predicted time to disease onset (7).

### Description of the Natural History of the Disease

**Figure 2:**
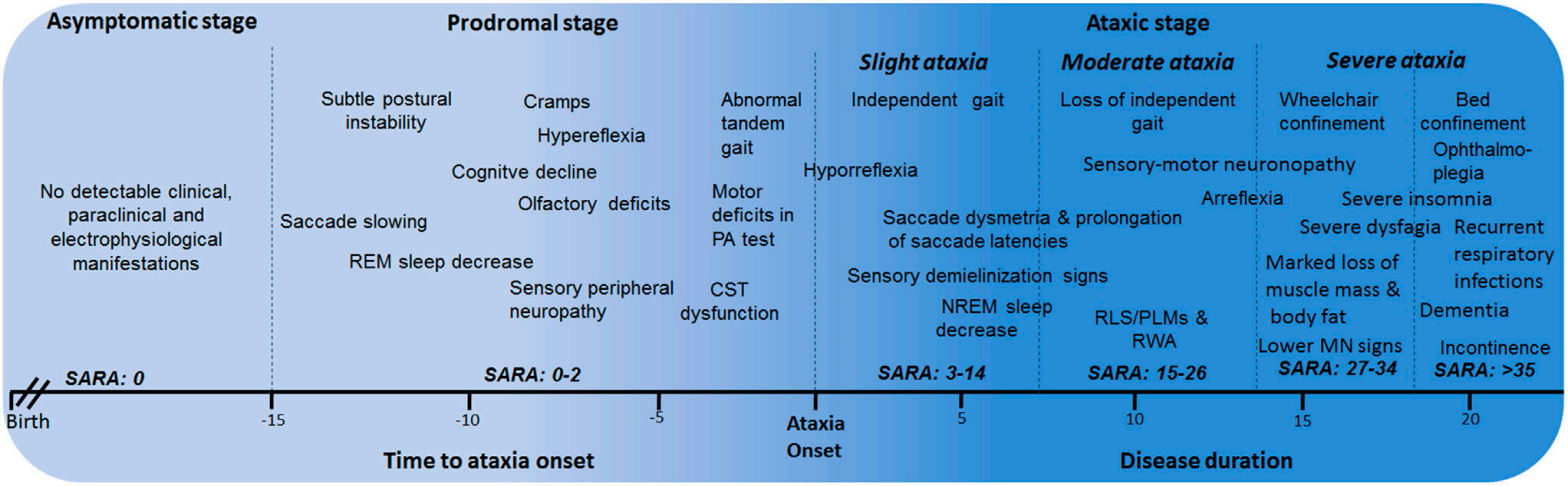
Stages of spinocerebellar ataxia type 2 (SCA2) progression. CST, corticospinal tract; PA, prism adaptation; REM, rapid eye movement; RLS, restless legs syndrome; PLMS, periodic leg movements syndrome; MN, motor neuron; RWA, REM sleep without atonia.

Based on all the phenotypical features of SCA2 since early disease stages, the natural history of SCA2 can be divided into the following major stages: asymptomatic, prodromal, and ataxic stages. The ataxic stage can also be subdivided according the severity of the cerebellar syndrome into slight ataxia, moderate ataxia, and severe ataxia (Figure 2). The description and characterization of these progression stages could be considered as model for other SCAs. The asymptomatic stage is characterized by the absence of detectable clinical, paraclinical, and neurophysiological disease features, and SARA scores 0. Following this stage, it is proposed the known prodromal stage, which is characterized by the beginning of first motor and non-motor abnormalities without a definite manifestation of cerebellar ataxia. This stage appears approximately 15 years before ataxia with the saccadic slowing, and REM sleep decreases as earliest SCA2 features (15). In prodromal stage, SARA scores range between 0 and 2 points (70).

Within the ataxic stage, the first period corresponds to the early manifestations of the cerebellar syndrome and independent gait (slight ataxia). It is characterized by the worsening of most of prodromal features, together with other emerging manifestations such as RLS/PLMS, sensory demyelization signs, among others. Usually, these cases exhibit SARA scores between 3 and 14 points, although a subgroup with scores among 3 and 8 points can be identified as early diagnosed subjects, which is the most appropriate cohort to enroll in future clinical trials.

The subsequent ataxic stage is associated to permanent support to walk (moderate ataxia) with SARA scores between 15 and 26 points as well as the worsening of non-motor features. After that, the wheelchair and bed confinement stages (severe ataxia) can be recognized. In the first of them, SARA score commonly varies between 27 and 34 points and the sensory-motor neuronopathy becomes evident, including marked loss of muscle mass and body fat, as well as notable difficulties to swallow. The bed confinement stage is characterized by the dramatic worsening of motor and non-motor features, SARA scores above 35 points, significant sensory conduction blockage, ophthalmoplegia, incontinence, severe insomnia, and important mental deficiencies resembling dementia in some cases as well as recurrent respiratory infections. Finally, the main causes of death are broncopneumonia, bronchial aspiration, and cardiovascular failures (6).

## Neuroanatomical Features

Macroscopic brain observations in post-mortem samples reveal a reduction of overall size of the brain with significant atrophy of the cerebellum, brainstem, frontal lobe, and cranial nerves. Moreover, whiteness of the midbrain substantia nigra and reduction of the cerebral and cerebellar white matter is detectable. Histopathological studies disclose an early and marked loss of neurons in the cerebellar Purkinje cell layer with reduction in the dendritic arborizations and torpedo-like deformations of axons. The granular cells as well as the parallel and climbing fibers are also sparse, whereas the dentate nucleus is relatively spared (4, 42). Changes in the brainstem include marked loss of inferior olive neurons, degeneration of pontine and other pre-cerebellar brainstem nuclei, as well as notable reduction of neurons of the substantia nigra in the mesencephalon. Widespread neuronal loss is also observed in cerebral cortex, basal forebrain, thalamus, and spinal cord (4, 42, 72, 73).

**Figure 3:**
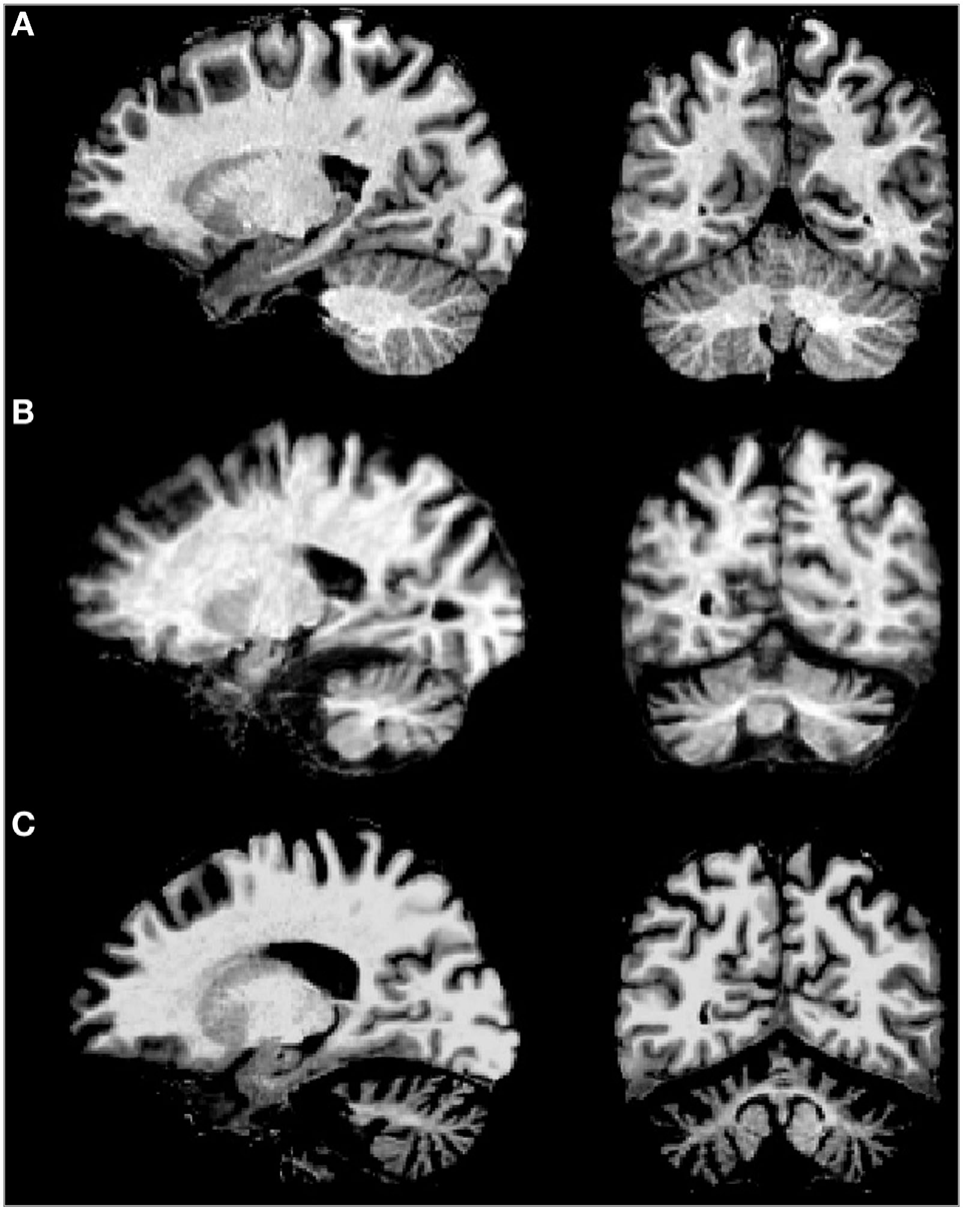
MRI of sagital (left) and coronal (right) examples of **(A)** normal brain, **(B)** spinocerebellar ataxia type 2 (SCA2) patient with early clinical manifestation, and **(C)** SCA2 patient with full ataxia manifestation. Note the severe cerebellar volume loss in gray and white matter in advanced stages of the disease.

Brain imaging techniques in SCA2 patients have revealed marked cerebellar volume loss in gray and white matter (Figure 3), as well as atrophy of the pons, medulla oblongata, spinal cord, parietal cortex, and thalamus (74, 75). MRI scans on the pontocerellar area suggest that the atrophy is not uniform but is region specific (76). Significant associations between brain imaging findings with motor and cognitive performance of SCA2 patients have been reported using Voxel-based morphometry and resting-state functional magnetic resonance imaging (77–79). In one example, SCA2 patients showing cognitive deficits, including spatial working memory, were found to have a significant degeneration of the parahippocampal gyrus (79).

Besides, a recent longitudinal Tensor-Based Morphometry Study conducted in 10 Italian SCA2 patients disclosed a significant progression of brain atrophy, in special in the substantia nigra, basis pontis, middle cerebellar peduncles cerebellar white matter, and cortical gray matter in the inferior portions of the cerebellar hemispheres (80).

Brain imaging markers in prodromal SCA2 reveal an early atrophy and/or dysfunction of pontocerebellar system. Inagaki et al. reported a significant decrease in glucose metabolism of this area in two out three preclinical carriers studied by PET (81). In addition, MRI volumetric analyses conducted in 50 preclinical carriers of SCA1, SCA2, SCA3, and SCA6 mutations from the EUROSCA cohort depicted greater brainstem atrophy in SCA2 as compared to other subtypes, while voxel-based morphometry assessments revealed a marked loss of cerebellar gray matter at lobules V and VI in SCA2 preclinical carriers (70).

Moreover, a MRI study in 24 Cuban SCA2 preclinical carriers identified the cerebellar atrophy (included vermis and hemispheres) as the most common finding (62.5%), followed by pons atrophy (29.2%), and light atrophy of the frontal cortex (20.8%). In this cohort, a marked association between the area of cerebellar vermis and the SARA score was reported (10).

## Genotypical Features

The SCA2 mutation consists on a unstable expansion of the CAG repeat tract in the 1st exon of the ATXN2 gene (12q23-q24.1). This repeat encodes a polyQ tract in the protein ataxin-2. Normal alleles vary from 13 to 31 triplet repeats, and alleles with 22 trinucleotide repeats are the most common [(CAG)_8_-CAA-(CAG)_4_-CAA-(CAG)_8_]. Alleles carrying 28–33 repeats are considered as intermediate expansions and may predispose to an elevated risk for ALS or the Parkinson plus syndrome PSP (82), ATXN2 expanded alleles present ≥32 triplet repeats with a large range of full penetrance above 35 repeats, which usually exhibit a pure CAG tract (83). The presence of CAA interruptions in expanded alleles appears to predispose to a phenotype with Parkinson or with motor neuron disease (37, 84, 85), although both CAG and CAA code for glutamine, indicating that the neuronal population affected by the pathogenesis is determined by RNA toxicity rather than protein toxicity.

The size of the expanded tract at the age at onset explains from 60 to 80% of its variance, suggesting the existence of modifier genes, genetic polymorphisms, epigenetic factors, and unknown environmental determinants modulating age of onset. Hence, the study of allelic association in individuals highly discordant for age of onset has identified the long normal CAG repeats in the *CACNA1A* (86) and *RAI1* genes (87), as well as the 10398G polymorphism in the mitochondrial complex gene (88) and the GSTO2 rs2297235 “AG” genotype (89), as modifier factors promoting an earlier manifestation age in SCA2 patients; nevertheless, some of these genetic modifiers need to be confirmed in distinct populations.

**Figure 4:**
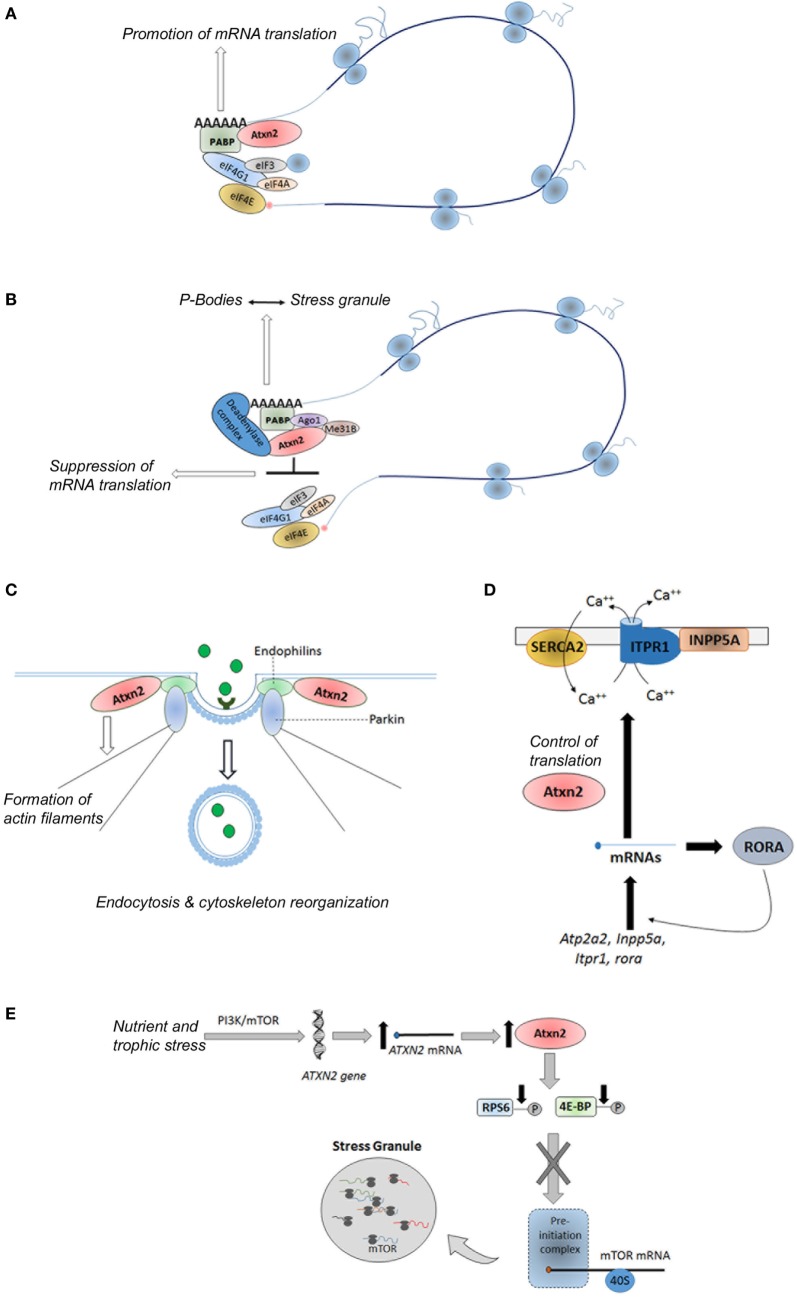
Physiological functions of ataxin-2. **(A)** Promotion of mRNA translation of specific genes via its interaction with the PABP and 3′ untranslated regions in the polyribosomes; **(B)** global suppression of translation under stress conditions via its interaction with the miRNA pathway proteins Ago1 and Me31b; **(C)** control of endocytosis through its binding to endophilins; **(D)** regulation of the calcium signaling pathway through the control of translation of some of their components; and **(E)** sensoring of nutritional and energetic state of the cells through the direct and/or indirect inhibition of the mTORC1 signaling pathway. Atxn2, Ataxin-2; PABP, polyA binding protein; eIF3, eukaryotic initiation factor 3; eIF4A, eukaryotic initiation factor4A; eIF4E, eukaryotic initiation factor 4E; IF4G1, eukaryotic initiation factor 4G1; Ago1, Argonuate 1; Me31b, Deadbox helicase me31B; SERCA2, smooth endoplasmic reticulum Ca-ATP-ase 2; INPP5A, inositol polyphosphate-5-phosphatase; Atp2a2, gene encoding the SERCA2 protein; RORA, retinoic acid-related orphan receptor alpha; PI3K, phosphoinositide 3-kinase; mTOR, mechanistic target of rapamycin; ITPR1, inositol triphosphate receptor 1; RPS6, ribosomal protein S6; 4E-BP, eIF4E-binding protein.

Ataxin-2 protein is a ubiquitously expressed polypeptide (90) involved in the regulation of several RNA processing pathways, endocytosis, modulation of calcium signaling pathways, as well as control of metabolism and energy balance (91–93) (Figure 4). Hence, ataxin-2 protein promotes the mRNAs translation of specific mRNAs through its binding to polyribosomes, polyA-binding protein (PABP) and to the 3′ untranslated regions (UTRs) of specific mRNAs (Figure 4A). As examples, the interaction between ataxin-2 and PABP regulates the translation of ataxin-3 in cellular models (94), as well as the expression of the *PERIOD* gene, involved in the circadian timing in *Drosophila melanogaster* (95). A subtle impact of ataxin-2 regulation of *PERIOD* gene in mammals was recently demonstrated in a Atxn2-KO mice (96). On the interaction between ataxin-2 and 3′ UTRs, Yokoshi et al. demonstrated the direct binding of this protein with the uridine-rich elements in the 3′ UTRs of its mRNA targets, which stabilize them and increase their translation (97).

Moreover, under stress conditions, ataxin-2 can suppress the translation of mRNAs leading the formation of stress granules through its interaction with the miRNA pathway components Me31B and Ago1 (Figure 4B) (98). Specifically, ataxin-2 regulates the translation of presynaptic and postsynaptic target mRNAs involved in the long-term olfactory habituation (99).

Moreover, studies in *Caenorhabditis elegans* and *D. melanogaster* have demonstrated a role of the ataxin-2 ortogue proteins in the reorganization of the cytoskeleton, the formation of actin filaments, and the assembly of endocytic vesicles through its binding to endophilins (Figure 4C) (100–103). In addition, recent findings reveal a role of the ataxin-2 in the regulation of the calcium signaling pathway through the control of translation of some of their components, such as *Atp2a2, Inpp5a, Itpr1*, and *rora* genes (104) (Figure 4D).

Recent evidence reveals that ATXN2 is involved in the complex network regulating peripheral and central signals related to food intake and body weight. In line with this function, ataxin-2 has been proposed as a nutritional and energetic sensor (90, 91), induced under cellular bioenergetics deficits, which hinders energy demanding anabolic processes through the direct and/or indirect inhibition of the mTORC1 signaling pathway (105, 106). The direct inhibition is based on the sequestration of mTOR transcripts into the stress granules, whereas indirect inhibition consists on the promoting of reduced phosphorylation of ribosomal protein S6 and eukaryotic initiation factor 4E-binding protein (Figure 4E) (105), two key substrates of the TORC1 complex (107).

Interestingly, it has been suggested that nuclear accumulation of ataxin-2 contributes to expanded ataxin-1-induced neurodegeneration in *D. melanogaster*, which reveals functional relationship between these proteins (108).

The presence of an expanded PolyQ tract in ataxin-2 tract likely causes conformational changes in ataxin-2 that result in gain and/or partial loss of function, leading to cellular dysfunction and neuronal cell death. Gain of toxic functions is usually associated to neuronal cell death, whereas the partial loss of function likely promotes metabolic disturbances.

Pathophysiological mechanisms underlying the gain of toxic functions of mutated ataxin-2 include toxic accumulation of cytoplasmatic protein aggregates, abnormal neuronal calcium signaling, abnormal protein recycling, proteolytic cleavage, transcriptional and translational dysregulation, and mitochondrial dysfunction (1, 109, 110). Recently, a mechanism of RNA toxicity was suggested for SCA2. This mechanism consists on the production of a neurotoxic anti-sense transcript (ATXN2-AS), which was detected in the post-mortem cerebellum and cortex of SCA2 patients, a transgenic mouse model and some cell lines (111).

## Biochemical Findings

Biochemical analyses of the cerebrospinal fluid (CSF) and blood plasma have become increasingly important for the physiopathological study and therapy design in SCA2. In these patients, a significant reduction of zinc levels both in CSF and serum has been documented, which seems to be caused by environmental deficits and unknown physiopathological mechanisms related to expanded CAG repeats (112). Moreover, SCA2 patients exhibit a notable reduction of erythropoietin in the CSF suggesting the dysfunction of endogenous neuroprotective mechanisms in the disease (6).

Measures of antioxidant-prooxidant balance in SCA2 patients suggest a significant increase in advanced oxidation protein products and peroxidation potential, as well as decrease in ferric reducing ability in plasma, GSH, and total hydroperoxides (113). Also, increased enzymatic activity was recently observed for the glutation *S*-transferase (GST) in a distinct cohort of Cuban patients (114). Furthermore, seminal biochemical studies in Cuban SCA2 patients demonstrated a significant reduction of dopamine and its metabolites in the CSF and detected a decreased concentration of ethanolamine, suggesting altered phospholipid metabolism (25). However, some of these biochemical alterations need to be confirmed in distinct populations as well as among patients with distinct clinical stages.

## Diagnosis

Unequivocal diagnosis of SCA2 must be established by molecular testing. However, when molecular testing is not possible or consented by the patient, a detailed family history and physical examination can provide some clinical suggestive findings that can lead to a diagnosis of SCA2; such is the case of severe slowing of horizontal saccades and low frequency of nystagmus (3).

Predictive testing for SCA2 has been available in Cuba for over 15 years to determine whether an at-risk individual inherited the expanded allele and to determine prenatally whether the fetus has inherited the expanded allele. Higher uptake rates, low frequency of severe mental health outcomes, and no catastrophic events distinguish these programs from others (115). Nevertheless, the higher frequency of large normal alleles in the Cuban population (19, 20) and the recently described role of large normal or intermediate *ATXN2* alleles in other neurodegenerative disorders (82) represent important challenges for the predictive testing and genetic counseling in SCA2.

## Therapeutic Options in SCA2

The therapeutic approaches for SCA2 are limited to supportive care that partially improves some cerebellar and non-cerebellar manifestations but fail to halt the progression of the disease (3, 6), as a result of some factors limiting the successful of effective clinical trials. First, SCA2 is still considered as rare disease, representing a not enough attractive disease for most pharmaceutical companies. Moreover, the majority of clinical trials have enrolled small and heterogeneous samples of subjects, most of them in advanced clinical stages of the disease, when the therapy is difficult by the extended neuronal degeneration. Another problem is the absence of predictive and progression biomarkers to assess the efficacy of therapeutic.

Till now, there are two kinds of therapeutic interventions that have been evaluated in SCA2 patients. These consist of pharmacological and physiotherapeutic interventions. A number of clinical trials have been assessed in SCA2 patients (summarized in Table 1), but in some of them, the findings need to be confirmed by means of future clinical trials, even in prodromal disease stages.

**Table 1:** Clinical trials in spinocerebellar ataxia type 2 patients.

Treatment	Doses	Type of trial	*n*	Follow-up (months)	Outcome
Lithium carbonate (116)	Starting with 150 mg BID until total daily dose of 1,500 mg or serum level of lithium (0.9–1.2 mEq/L)	Double blind, placebo	16	12	Non-significant changes in SARA and brain volume. Significant reduction in the BDI
Riluzole (117)	100 mg daily	Double blind, placebo	16	12	Decrease of SARA score
Zinc sulfate (112)	50 mg daily	Double blind, placebo	36	6	Improved gait, posture, stance, dysdiadochocinesia, and reduction of saccadic latency
Lisuride (118)	0.1 mg daily 1 h before bedtime	Open label	12	1	Decrease in PLMS index and improved subjective sleep quality
B vitamins (119, 120)	10,000 U/weeks during first 4 weeks and 5,000 U/weeks until 12th week	Open label	20	3	Improved sensory neuropathy and painful muscle cramps

*SARA, Scale for Assessment and Rating of Ataxia; BDI, Beck’s depression inventory; PLMS, periodic leg movement syndrome*.

For example, a recent 1-year clinical trial in 16 SCA2 patients using lithium carbonate that was safe and well tolerated by the participants failed to show any effective changes in the ataxia severity (116). In 2015, Romano and coworkers (117) published a randomized, double-blind, placebo-controlled pilot trial with riluzole. The clinical trial was conducted in a mixed sample of hereditary ataxia including 16 SCA2 patients, with the aim to reduce the cerebellar hyper-excitability by means the activation of some anti-glutamatergic neuroprotective mechanisms such as the activation of small-conductance calcium-activated potassium channels, the inhibition of glutamate presynaptic release, the promotion astrocytes-dependent glutamate clearance from synaptic clefts, as well as the stimulation of neurotrophic factors synthesis. Although some efficacy of riluzole on the ataxia severity was demonstrated, the reduced sample sizes in this study encourage the need to confirm this finding in a large and homogeneous cohort of patients.

The large and homogeneous population of Cuban SCA2 patients have underwent various clinical trials. In 2006, Velázquez-Pérez and coworkers reported the findings on a 6-month double-blinded and placebo-controlled clinical trial with 50 mg zinc sulfate in 36 Cuban SCA2 patients. This study demonstrated the efficacy of this supplementary treatment on the zinc dyshomeostasis and its subtle effects on cerebellar syndrome, peripheral neuropathy, saccade pathology, and oxidative stress (112).

Moreover, a pilot study using lisuride (0.1 mg/day during 4 weeks) demonstrated the efficacy of this dopaminergic treatment on the PLMS and other sleep disturbances (118). In addition, an open clinical trial conducted in 20 Cuban patients using high doses of B-complex vitamins showed significantly improvements of the clinical and electrophysiological markers of peripheral neuronopathy. Furthermore, a significant decrease of painful muscle cramps was found in 53% of the cases after treatment, as well as a partial recovery of cognitive alterations (119, 120).

Recent findings based on the reduction of ataxin-2 expression by means anti-sense oligonucleotide (ASO) therapy revealed the efficacy of this therapeutical approach in the motor performance and Purkinje cells firing rate in SCA2 mouse models (121), which represents promising options for future clinical trials in humans. Interestingly, the ASO therapy targeting ataxin-2 reduced the disease pathology and increased the animal’s life spam in an ALS mouse model (122).

Physiotherapy is a very important strategy in the rehabilitation of SCA2 patients. Rodríguez-Díaz et al. (123) performed a study in 96 SCA2 Cuban patients who were treated 6 h daily during 2 months showing a significant improvement in coordination, postural stability, saccade latency, and antioxidant defenses, supported by the increase in the enzymatic activity of the GST. In addition, Pérez-Avila et al. (124) observed a significant improvement of posture and coordination in 87 SCA2 subjects who underwent an exercise-training program during 6 months.

## Conclusion and Future Directions

Over almost 30 years, a large number of studies have been conducted in order to understand the SCA2 phenotype and its relationship with the genotype in some populations, especially in the large and homogeneous population of Cuban SCA2 families. These efforts have allowed a comprehensive characterization of distinct disease stages emphasizing in the recently described prodromal stage, which set a promising scenario to future therapeutical trials since in these early stages the neurodegeneration is still incipient.

Herein, in spite this valuable knowledge on SCA2, much more still needs to be learned regarding the genotype-phenotype relationship, the physiopathological basis of the neurodegeneration, and the prodromal stage characterization. Also, the beginning of treatments in preclinical subjects in conjunction with the presymptomatic testing results in important ethical concerns that need to be appropriately addressed in the clinical practice. Other key issues deserving special attention in future researches are the pathogenetic link between *ATXN2* intermediate expansions and other neurodegenerative disease, such as ALS, which challenges the genetic testing for CAG repeat expansions in this gene as patients with ataxia and a family history of ALS.

## Search Strategy and Selection Criteria

We searched PubMed and SCOPUS for papers published between January 1, 2000, and July 28, 2016, with the following terms: “spinocerebellar ataxia type 2,” “SCA2,” “SCA2 phenotype,” “SCA2 genetics,” “SCA2 diagnosis,” “SCA2 treatments,” “preclinical stage,” “prodromal stage,” “presymptomatic stage,” “mutation carriers,” “preclinical carriers,” and “presymptomatic subjects.” We used no language restrictions. The final reference list was generated based on relevance to the topics covered in this review.

## Author Contributions

LV-P and RR-L did the literature search and review. LV-P, RR-L, and JR contributed to editing and writing of the full review. No medical writer or editor was involved in the creation of the manuscript.

## Conflict of Interest Statement

The authors declare that the research was conducted in the absence of any commercial or financial relationships that could be construed as a potential conflict of interest.
